# Epigenomic profiling links IGF2 overexpression to anthracycline resistance in colorectal cancer organoids

**DOI:** 10.1172/jci.insight.195194

**Published:** 2026-07-16

**Authors:** Tara L. Hogenson, William J. Phillips, Merih D. Toruner, Zachry S. Poshusta, Luciana L. Almada, Hao Xie, Ryan M. Carr, Jenny J. Li, David L. Marks, Renzo E. Vera, Erik Jessen, Michael T. Barrett, Joleen M. Hubbard, Travis E. Grotz, Martin E. Fernandez-Zapico

**Affiliations:** 1 Division of Oncology Research,; 2 Division of Medical Oncology, and; 3 Division of Computational Biology, Mayo Clinic, Rochester, Minnesota, USA.; 4 Division of Hematology/Oncology, Mayo Clinic, Phoenix, Arizona, USA.; 5 Department of Surgery, Mayo Clinic, Rochester, Minnesota, USA.

**Keywords:** Gastroenterology, Oncology, Therapeutics

## Abstract

This study provides evidence of a new treatment approach for colorectal peritoneal metastases, a condition associated with a worse prognosis than other types of metastases.

Colorectal cancer (CRC) peritoneal metastases (CPMs) are associated with increased chemotherapy resistance and reduced overall survival compared with other types of CRC metastases (1). Consequently, there is a critical need to better understand the mechanisms driving therapeutic responses in CPMs to improve patient outcomes. We developed a cohort of CPM patient–derived organoids (PDOs) from patients undergoing cytoreductive surgery using our published protocol (2). We successfully grew PDOs from 54% of our CPM specimens for 3 or more passages (Supplemental Figure 1A; supplemental material available online with this article; https://doi.org/10.1172/jci.insight.195194DS1). Exome sequencing was performed on PDOs, as well as a subset of matched primary tumor and CPMs, with a high correlation rate of 94% between the primary tumor, CPM, and PDO (Supplemental Figure 1B). We identified common pathogenic variants in well-known CRC drivers (e.g., *APC*, *PIK3CA*, *KRAS*, *TP53*) and less common CRC pathogenic mutations (e.g., *BRCA1*, *LRP1B*, *IDH1*) (Supplemental Figure 1C). Immunohistochemical analysis of a primary tumor, CPM, and corresponding PDO showed distinct morphological features and marker expression patterns common to CRC (Supplemental Figure 1D). RNA-seq analysis found the majority of PDOs had low levels of colonic stem cell markers (Supplemental Figure 1E).

We assessed CPM PDO response to 3 commonly used CRC CPM chemotherapies (1), doxorubicin, mitomycin C (MMC), and oxaliplatin (Figure 1A and Supplemental Figure 2, A–C). Responses to MMC and oxaliplatin were heterogeneous, whereas most PDOs were sensitive to doxorubicin except HO199, derived from patient 6 (Figure 1B). Based on these findings, we selected a subset of CPM PDOs to investigate mechanisms underlying doxorubicin response.

Assay for transposase-accessible chromatin with sequencing (ATAC-seq) identified over 4,000 regions with increased accessibility in PDOs from patient 6 (Supplemental Figure 2, D and E), while RNA-seq revealed enrichment of a subset of genes (Supplemental Figure 2, F and G). Integrative epigenomic and transcriptomic analysis identified gene targets in patient 6 PDOs, with insulin-like growth factor 2 (*IGF2*) as the top predicted target (Figure 1, C and D), which is known to play a role in CRC carcinogenesis (3, 4). Increased IGF2 mRNA and protein expression, along with its associated pathway markers, were confirmed in patient 6 PDOs (Supplemental Figure 2, H–J).

To define the therapeutic significance, we tested whether inhibition of IGF signaling using the selective IGF1 receptor (IGF1R) inhibitor linsitinib could restore doxorubicin sensitivity. Increased IGF2 expression was associated with enhanced sensitivity to linsitinib (Figure 1E), and combining doxorubicin with linsitinib decreased viability in our IGF2-high CPM PDOs (Figure 1, F and G). To determine whether this effect extended to other anthracyclines, we evaluated responses to daunorubicin and idarubicin. Like doxorubicin, PDO HO199 remained the most resistant to single-agent treatment (Supplemental Figure 3, A and B). Cotreatment with linsitinib reduced viability in IGF2-high PDOs treated with either anthracycline (Supplemental Figure 3, C and D). Highest Single Agent (HSA) analysis showed significantly decreased viability in one or more IGF2-high PDOs when treated with an anthracycline plus linsitinib relative to HO178 (Figure 1H). Like linsitinib, targeting IGF1R using a monoclonal antibody, ganitumab, produced a combinatorial effect when combined with doxorubicin (Supplemental Figure 3, E–G). Targeting efficacy was measured using phosphorylated AKT (p-AKT) following treatment with linsitinib or ganitumab. HO199 showed decreased p-AKT expression, supporting stronger pathway inhibition in the IGF2-high PDOs (Supplemental Figure 3, H and I). Neutralization of IGF2 with a blocking antibody caused a small reduction in viability when combined with doxorubicin, and no effect as a single agent (Supplemental Figure 3, J–L).

Notably, this combination effect was specific to anthracyclines, as no significant increase in sensitivity was observed when linsitinib was combined with MMC or oxaliplatin (Figure 1H and Supplemental Figure 4, A and B). To determine whether this effect was specific to CPMs, we evaluated doxorubicin response and *IGF2* mRNA expression in 6 PDOs derived from CRC liver metastases (CLMs) (2). We identified 2 doxorubicin-resistant CLM PDOs (Supplemental Figure 4C) with either high (HO12) or low (HO98) *IGF2* expression (Supplemental Figure 4, D and E). Like the CPM PDOs, the *IGF2*-high PDO HO12 showed increased doxorubicin sensitivity when combined with linsitinib (Supplemental Figure 4, F and G).

While IGF1R-targeted therapies have previously shown disappointing results in clinical trials, many of these studies did not stratify patients by IGF2 expression, which is commonly upregulated in CRC and may serve as a biomarker of therapeutic response (4, 5). Our findings show that IGF1R inhibition in CRC PDOs with high IGF2 expression restores anthracycline sensitivity, suggesting a therapeutic vulnerability that could improve response in metastatic CRC. Finally, our findings demonstrate that CRC PDOs provide a valuable platform for comprehensive molecular profiling and functional testing of combination therapies, enabling the identification of precision treatment strategies to help improve patient outcomes.

## Conflict of interest

The authors have declared that no conflict of interest exists.

## Funding support

This work is the result of NIH funding, in whole or in part, and is subject to the NIH Public Access Policy. Through acceptance of this federal funding, the NIH has been given a right to make the work publicly available in PubMed Central.

- NIH grant CA265050.
- Joan F. & Richard A. Abdoo Family Fund in Colorectal Cancer Research.
- GI Cancer program of the Mayo Clinic Cancer Center (Mayo Clinic).
- Center of Individualized Medicine (Mayo Clinic).
- Department of Laboratory Medicine and Pathology (Mayo Clinic).
- Minnesota Colorectal Cancer Research Foundation.

## Supplementary Material

Supplemental data

Unedited blot and gel images

Supporting data values

## Figures and Tables

**Figure 1:**
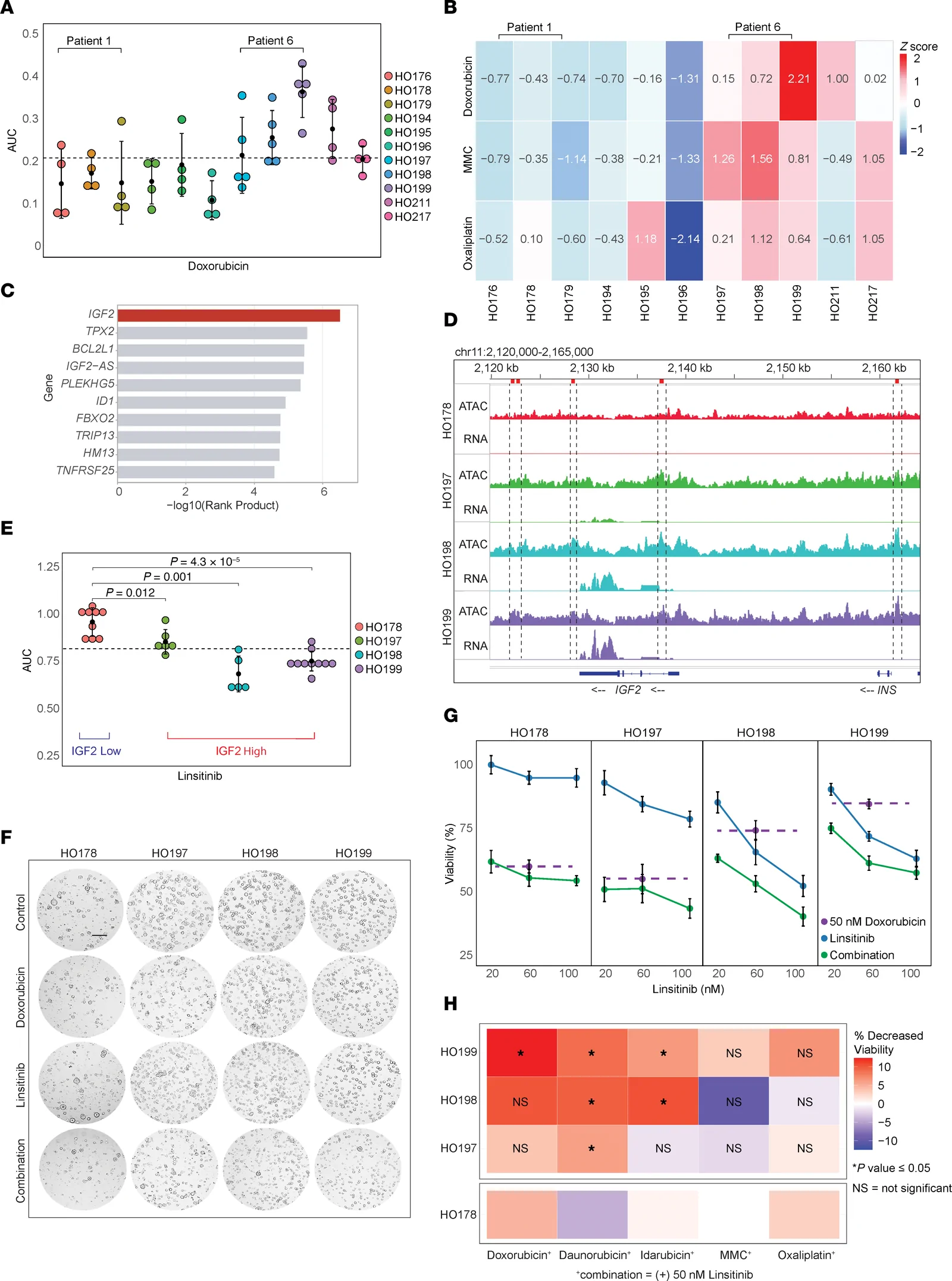
Treatment with an IGF1R inhibitor sensitizes IGF2-overexpressing CPM PDOs to doxorubicin. (**A**) Dot plot of doxorubicin area under the curve (AUC) for 11 CPM PDOs (derived from Supplemental Figure 2A). (**B**) *Z*-score heatmap of AUC for 11 CPM PDOs treated with doxorubicin, MMC, and oxaliplatin (higher *Z*-score = resistant). (**C**) Binding and Expression Target Analysis (BETA) of RNA-seq and ATAC-seq identifying top transcriptional targets in patient 6 CPM PDOs (–log_10_[rank product]). (**D**) ATAC-seq and RNA-seq signals at the *IGF2* locus; dashed lines indicate regions of significantly increased chromatin accessibility in patient 6 CPM PDOs (DiffBind). (**E**) Dot plot of linsitinib AUC for 4 CPM PDOs (derived from linsitinib response in **G**). (**F**) Bright-field images of 4 CPM PDOs treated with vehicle (control), doxorubicin (50 nM), linsitinib (50 nM), or a combination at 6 days. Scale bar: 250 μm. (**G**) Percentage viability of 4 CPM PDOs treated with doxorubicin (50 nM fixed), linsitinib (10, 50, 100 nM), or a combination. (**H**) Highest Single Agent (HSA) heatmap showing percentage decrease in viability (HSA minus combination). Positive values (red) indicate the combination outperformed single agent; negative values (blue) indicate HSA outperformed the combination. *P* values from pairwise Wilcoxon rank-sum tests versus HO178 (IGF2-low; **E** and **H**). NS, not significant. Black dots = sample mean; dashed line = cohort mean (**A** and **E**). Data are presented as mean ± SEM.

## Version 1 07/20/2026

In-Press Preview

## Version 2 08/24/2026

Electronic publication
