# Supplementary material for: Epigenomic profiling links IGF2 overexpression to anthracycline resistance in colorectal cancer organoids
Source: JCI Insight. 2026 Jul 16;11(16):e195194. doi: 10.1172/jci.insight.195194 (PMC13505381; doi:10.1172/jci.insight.195194)
Supplement: Supplementary file 1 [file jciinsight-11-195194-s002.pdf]

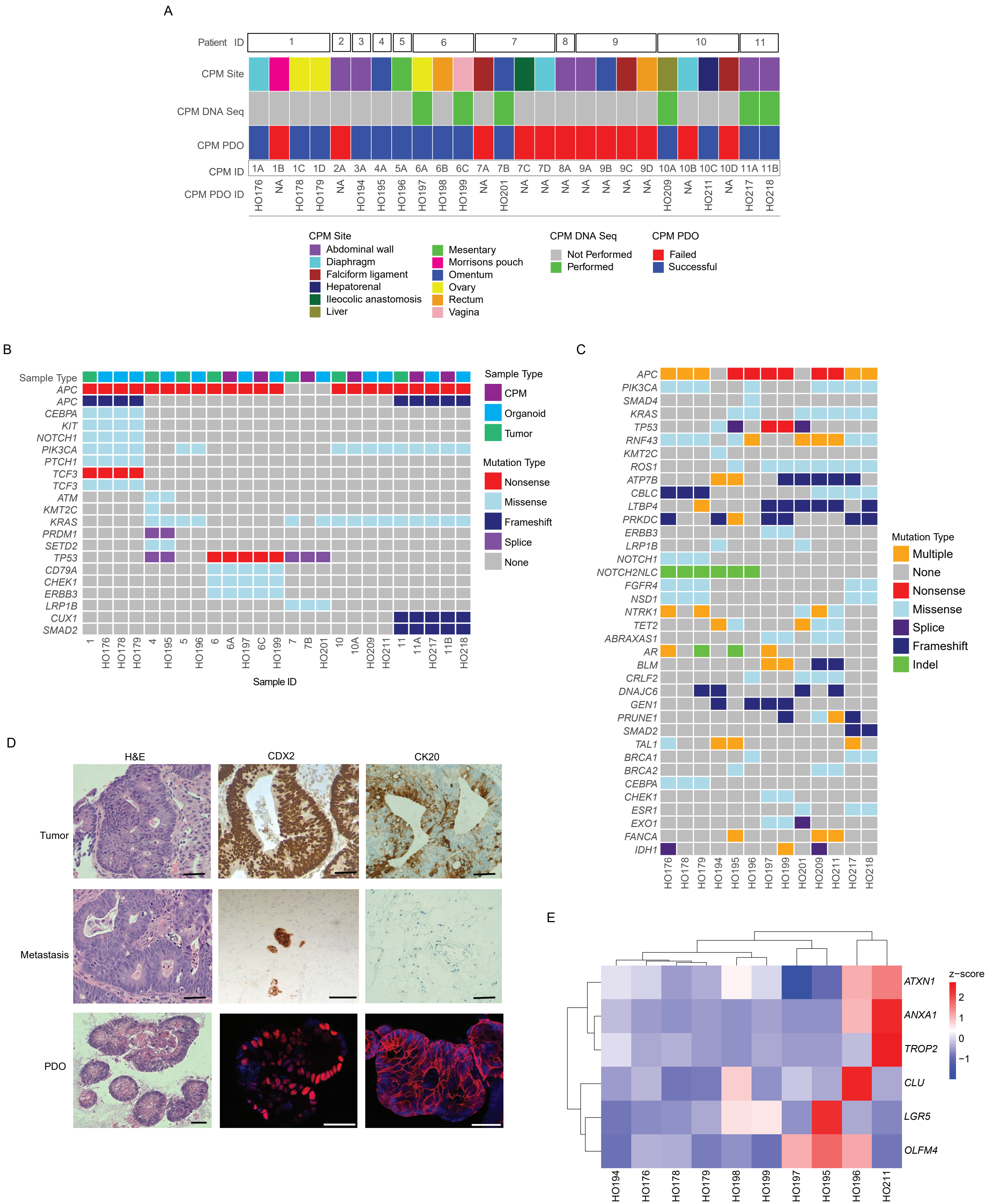
A
Patient ID
1
2
3
4
5
6
7
8
9
10
11
CPM Site
CPM DNA Seq
CPM PDO
CPM ID
1A 1B 1C 1D 2A 3A 4A 5A 6A 6B 6C 7A 7B 7C 7D 8A 9A 9B 9C 9D 10A 10B 10C 10D 11A 11B
CPM PDO ID
HO176 NA HO178 HO179 NA HO194 HO195 HO196 HO197 HO198 HO199 NA HO201 NA NA NA NA NA NA NA HO209 NA HO211 NA HO217 HO218
CPM Site
Abdominal wall
Diaphragm
Falciform ligament
Hepatorenal
Ileocolic anastomosis
Liver
Mesentary
Morrisons pouch
Omentum
Ovary
Rectum
Vagina
CPM DNA Seq
Not Performed
Performed
CPM PDO
Failed
Successful
B
Sample Type
APC
APC
CEBPA
KIT
NOTCH1
PIK3CA
PTCH1
TCF3
TCF3
ATM
KMT2C
KRAS
PRDM1
SETD2
TP53
CD79A
CHEK1
ERBB3
LRP1B
CUX1
SMAD2
1 HO176 HO178 HO179 4 HO195 5 HO196 6 6A HO197 6C HO199 7 7B HO201 10 10A HO209 HO211 11 11A HO217 11B HO218
Sample ID
Sample Type
CPM
Organoid
Tumor
Mutation Type
Nonsense
Missense
Frameshift
Splice
None
C
APC
PIK3CA
SMAD4
KRAS
TP53
RNF43
KMT2C
ROS1
ATP7B
CBLC
LTBP4
PRKDC
ERBB3
LRP1B
NOTCH1
NOTCH2NLC
FGFR4
NSD1
NTRK1
TET2
ABRAXAS1
AR
BLM
CRLF2
DNAJC6
GEN1
PRUNE1
SMAD2
TAL1
BRCA1
BRCA2
CEBPA
CHEK1
ESR1
EXO1
FANCA
IDH1
HO176 HO178 HO179 HO194 HO195 HO196 HO197 HO199 HO201 HO209 HO211 HO217 HO218
Mutation Type
Multiple
None
Nonsense
Missense
Splice
Frameshift
Indel
D
H&E
CDX2
CK20
Tumor
Metastasis
PDO
E
ATXN1
ANXA1
TROP2
CLU
LGR5
OLFM4
z-score
2
1
0
−1
HO194 HO176 HO178 HO179 HO198 HO199 HO197 HO195 HO196 HO211

Supplemental Figure 1. CPM PDOs are representative of the donor metastatic tissue.

A) Summary of CRC CPM PDOs including patient ID, site of metastasis within the peritoneal cavity, Exome sequencing availability for matched metastatic tissue, PDO generation success from CPM tissue, CPM ID based on patient ID and number of samples collected from the patient, and PDO ID assigned to CPMs that successfully generated organoids. PDOs were successfully generated from 9 patients, including PDOs from multiple independent lesions for patients 1, 6, 10, and 11. B) Oncoplot comparing mutation status between the primary tumor, metastases, and PDOs from the same patient. C) Mutational landscape of CPM PDOs for genes associated with CRC carcinogenesis including disease drivers such as *APC*, *PIK3CA*, *SMAD4,* and *KRAS*. D) Brightfield images of H&E stains of the primary tumor, metastasis 1A, and PDO HO176 from patient 1. Images also include IHC for CDX2 and CK20 in the primary tumor and metastasis. Confocal IF of the same markers is shown in the PDOs where red = CDX2 or CK20 and blue = DAPI (Scale bar = 50 μm). E) Heatmap showing the z-score for RNA-seq mRNA expression of colonic stem cell markers in CPM PDOs.

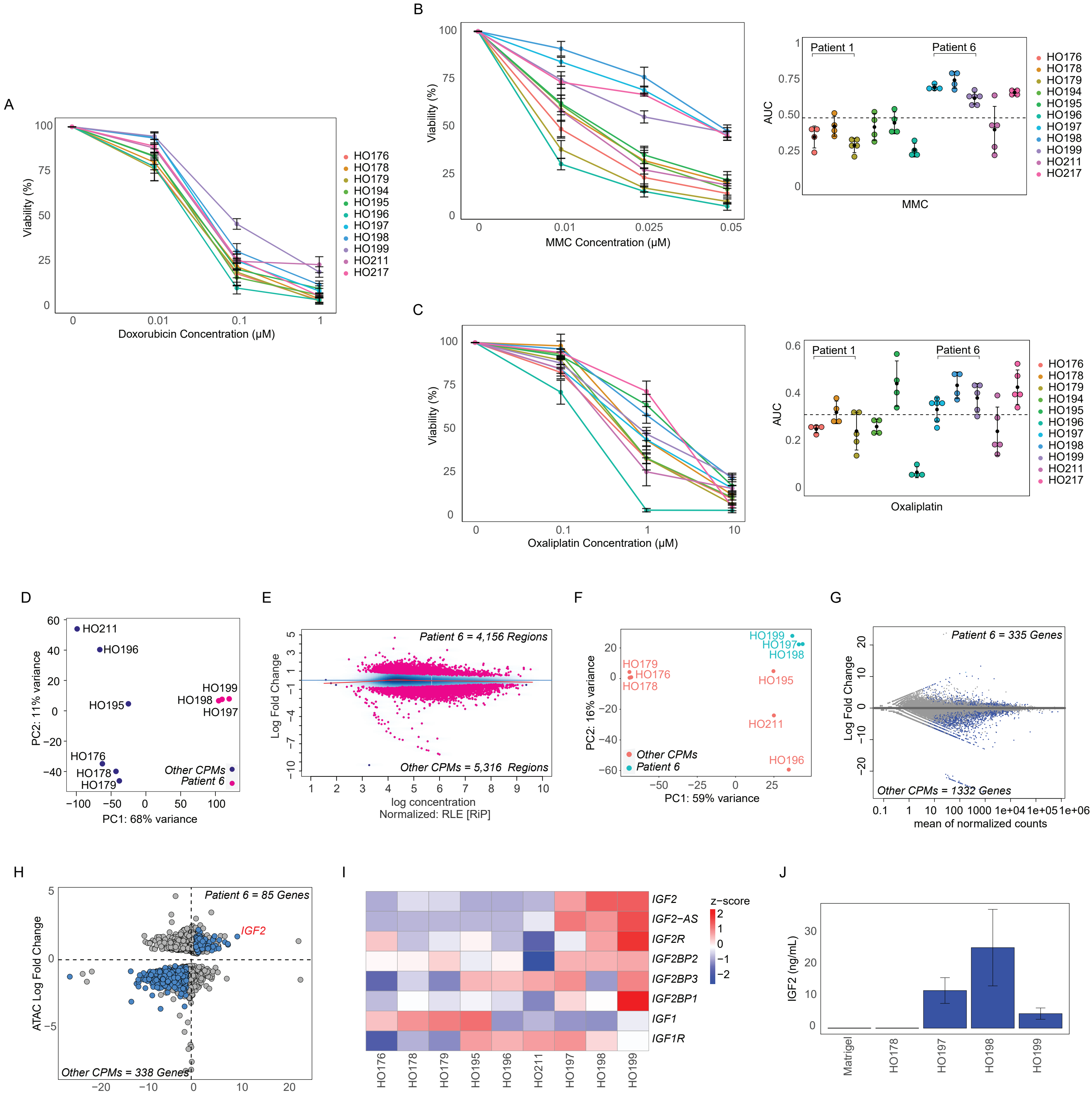
A
Viability (%)
Doxorubicin Concentration (µM)
0
0.01
0.1
1
100
75
50
25
HO176
HO178
HO179
HO194
HO195
HO196
HO197
HO198
HO199
HO211
HO217
B
Viability (%)
MMC Concentration (µM)
0.025
0.05
AUC
Patient 1
Patient 6
MMC
0.75
0.50
0.25
C
Viability (%)
Oxaliplatin Concentration (µM)
10
AUC
Oxaliplatin
0.6
0.4
0.2
D
PC2: 11% variance
PC1: 68% variance
Other CPMs
Patient 6
E
Patient 6 = 4,156 Regions
Other CPMs = 5,316 Regions
Log Fold Change
log concentration
Normalized: RLE [RiP]
F
PC2: 16% variance
PC1: 59% variance
Other CPMs
Patient 6
G
Patient 6 = 335 Genes
Other CPMs = 1332 Genes
Log Fold Change
mean of normalized counts
H
Patient 6 = 85 Genes
Other CPMs = 338 Genes
IGF2
ATAC Log Fold Change
RNA Log Fold Change
I
IGF2
IGF2−AS
IGF2R
IGF2BP2
IGF2BP3
IGF2BP1
IGF1
IGF1R
z−score
J
IGF2 (ng/mL)
Matrigel
HO178
HO197
HO198
HO199

Supplemental Figure 2. IGF2 upregulation is associated with doxorubicin resistance.

A) Line graph showing percent viability for 11 CPM PDOs in response to doxorubicin. B) Line graph and dot plot showing percent viability and relative area under the curve (AUC) for 11 CPM PDOs in response to MMC. C) Line graph and dot plot showing percent viability and relative AUC for 11 CPM PDOs in response to oxaliplatin. D) ATAC-seq PCA plot comparing patient 6 CPM PDOs (n=3) to the other CPM PDOs (n=6). E) MA plot showing differentially accessible regions between patient 6 CPM PDOs (n=3) and the other CPM PDOs (n=6) using DiffBind with RLE normalization. Pink dots are regions with a significant increase in accessibility with an FDR ≤ 0.05. F) RNA-seq PCA plot comparing patient 6 CPM PDOs (n=3) and other CPM PDOs (n=6). G) MA plot showing differentially expressed genes between patient 6 CPM PDOs (n=3) and other CPM PDOs (n=6) using DeSeq2. Genes highlighted in blue have an FDR ≤ 0.05 and log2fold change <-1 or >1. H) Integration of log fold change data for ATAC-seq (y-axis) and RNA-seq (x-axis). Genes highlighted in blue are significantly altered in both assays with an FDR ≤ 0.05. I) Heatmap showing the z-score for RNA-seq expression of *IGF2* and associated genes across all CPM PDOs. J) Protein levels of IGF2 in ng/mL in a subset of CPM PDOs using ELISA (n=3); matrigel included as a control. Each dot in the AUC graph = one replicate for each PDO derived from the neighboring line graph; black dots = sample mean; dashed line = cohort mean (panels B and C). Error bars = ± SEM.

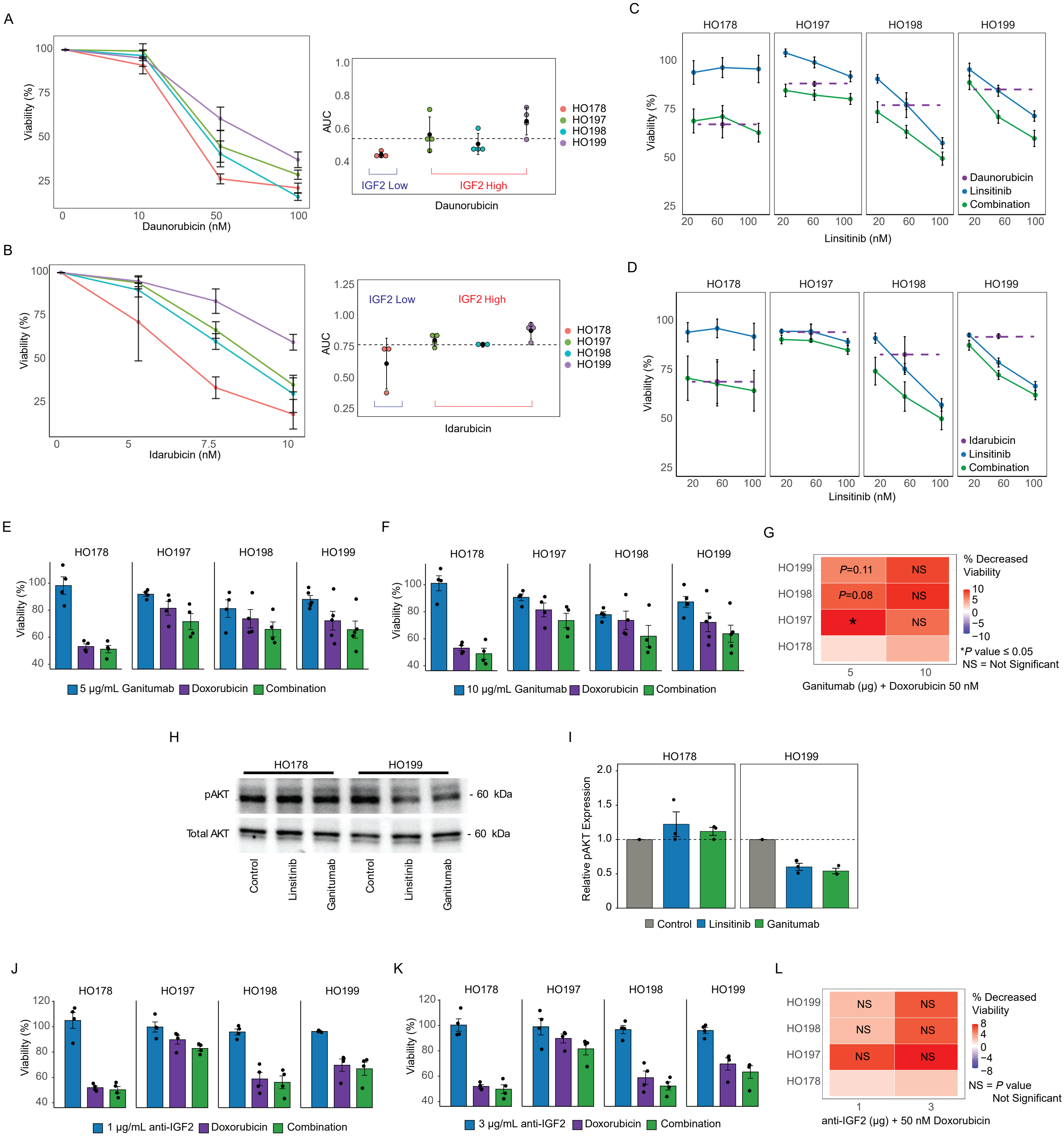
A
Viability (%)
100
75
50
25
0
10
50
100
Daunorubicin (nM)
AUC
1.0
0.8
0.6
0.4
IGF2 Low
IGF2 High
Daunorubicin
HO178
HO197
HO198
HO199
B
Idarubicin (nM)
5
7.5
1.25
1.00
0.75
0.50
0.25
Idarubicin
C
Linsitinib (nM)
20
60
Daunorubicin
Linsitinib
Combination
D
Idarubicin
E
5 μg/mL Ganitumab
Doxorubicin
F
10 μg/mL Ganitumab
G
% Decreased Viability
P=0.11
P=0.08
NS
*
*P value ≤ 0.05
NS = Not Significant
Ganitumab (μg) + Doxorubicin 50 nM
H
pAKT
Total AKT
- 60 kDa
Control
Ganitumab
I
Relative pAKT Expression
2.0
1.5
1.0
0.5
Control
J
1 μg/mL anti-IGF2
K
3 μg/mL anti-IGF2
L
anti-IGF2 (μg) + 50 nM Doxorubicin
NS = P value Not Significant

Supplemental Figure 3. Treatment with an IGF1R inhibitor sensitizes CPM PDOs to anthracyclines. A) Line graph and dot plot showing percent viability and relative AUC for 4 CPM PDOs in response to daunorubicin. B) Line graph and dot plot showing percent viability and relative AUC for 4 CPM PDOs in response to idarubicin. C) Line graph showing percent viability of 4 CPM PDOs in response to daunorubicin (25 nM fixed), linsitinib (10, 50, 100 nM), or a combination. D) Line graph showing percent viability of 4 CPM PDOs in response to idarubicin (6 nM fixed), linsitinib (10, 50, 100 nM), or a combination. E, F) Bar graph showing percent viability of 4 CPM PDOs in response to 50 nM doxorubicin, 5 µg/mL ganitumab (E), 10 µg/mL ganitumab (F), or a combination. Each black dot = one replicate. G) HSA heatmap showing percent decrease in viability (HSA – combination). Positive values (red) indicate the combination outperformed single agent; negative values (blue) indicate HSA outperformed the combination. H) PDO protein expression levels for phosphorylated AKT S473 (p-AKT) and total AKT following 6-hour treatment with 50 nM linsitinib or 10 µg/mL ganitumab (n=3). I) Quantification of PDO protein expression levels for p-AKT and total AKT following treatment as shown in panel H (n=3). p-AKT signal normalized to total AKT; vehicle control set to 1. J, K) Bar graph showing percent viability of 4 CPM PDOs in response to 50 nM doxorubicin, 1 µg/mL anti-IGF2 (J), 3 µg/mL anti-IGF2 (K), or a combination. Each black dot = one replicate. L) HSA heatmap showing percent decrease in viability (HSA – combination). *P* values from pairwise Wilcoxon rank-sum tests versus HO178 (IGF2-low); NS = Not Significant (panels G and L). Each dot in the AUC graph = one replicate for each PDO derived from the neighboring line graph; black dots = sample mean; dashed line = cohort mean (panels A and B). Error bars = ± SEM.

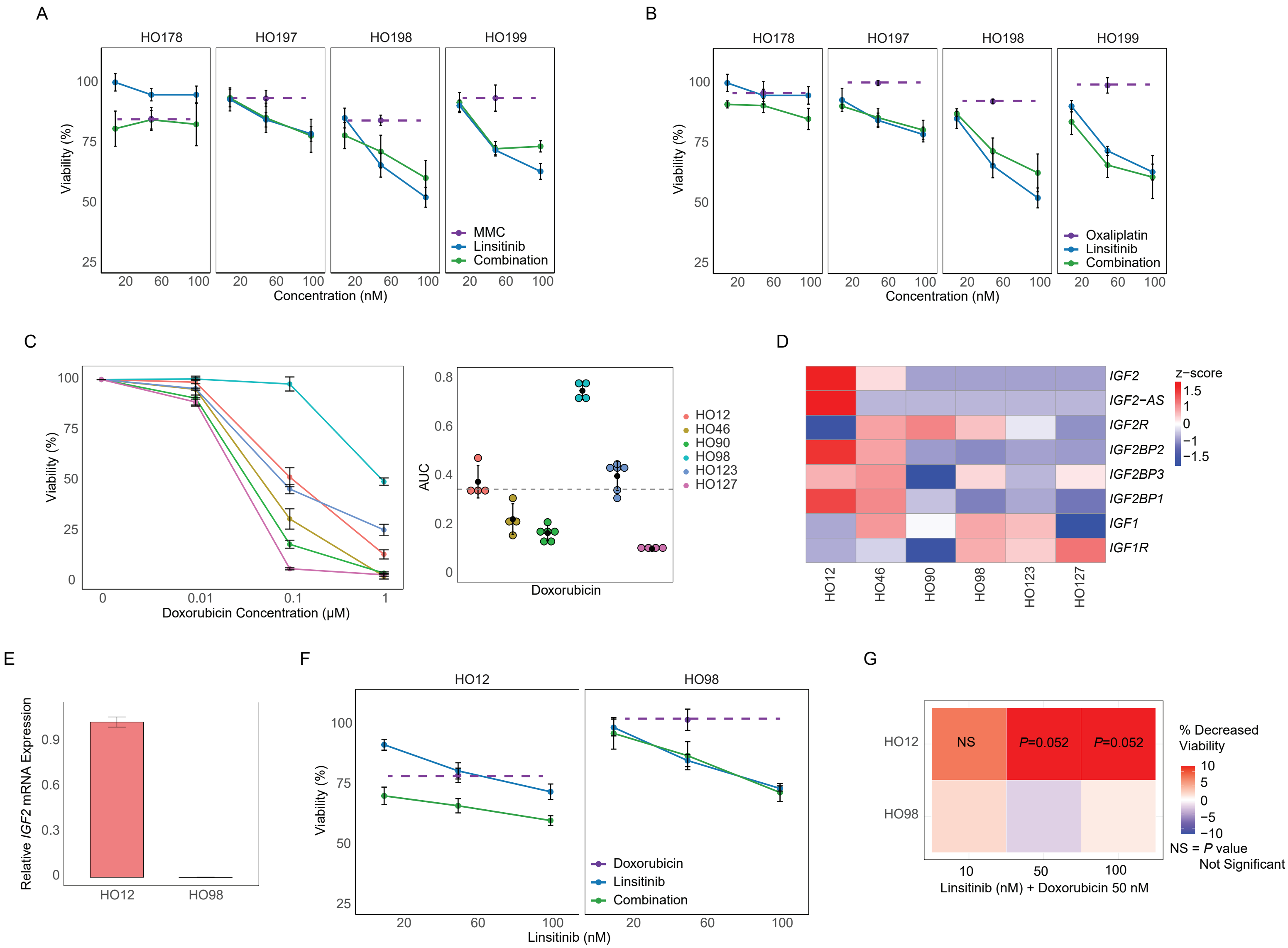

A
HO178
HO197
HO198
HO199
Viability (%)
Concentration (nM)
MMC
Linsitinib
Combination
B
Oxaliplatin
Linsitinib
Combination
C
Viability (%)
Doxorubicin Concentration (μM)
AUC
Doxorubicin
HO12
HO46
HO90
HO98
HO123
HO127
D
IGF2
IGF2−AS
IGF2R
IGF2BP2
IGF2BP3
IGF2BP1
IGF1
IGF1R
z−score
E
Relative IGF2 mRNA Expression
HO12
HO98
F
Viability (%)
Linsitinib (nM)
Doxorubicin
Linsitinib
Combination
G
NS
P=0.052
P=0.052
% Decreased Viability
NS = P value Not Significant
Linsitinib (nM) + Doxorubicin 50 nM

Supplemental Figure 4. Treatment with linsitinib is specific to anthracyclines and sensitizes CLM PDOs to doxorubicin. A) Line graph showing percent viability of 4 CPM PDOs in response to MMC (10 nM fixed), linsitinib (10, 50, 100 nM), or a combination. B) Line graph showing percent viability of 4 CPM PDOs in response to oxaliplatin (250 nM fixed), linsitinib (10, 50, 100 nM), or a combination. C) Line graph and dot plot showing percent viability and relative AUC of 6 CLM PDOs in response to doxorubicin. Each dot in the AUC graph = one replicate for each PDO derived from the neighboring line graph; black dots = sample mean; dashed line = cohort mean. D) Heatmap showing the z-score for RNA-seq expression of *IGF2* and associated genes across 6 CLM PDOs. E) mRNA expression of *IGF2* relative to *TBP* and *GAPDH* in two CLM PDOs, HO12 and HO98 (n=3). F) Line graph showing percent viability of two CLM PDOs in response to doxorubicin (50 nM fixed), linsitinib (10, 50, 100 nM) or a combination. G) HSA heatmap showing percent decrease in viability (HSA – combination). Positive values (red) indicate the combination outperformed single agent; negative values (blue) indicate HSA outperformed the combination. *P* values from pairwise Wilcoxon rank-sum tests comparing mean % decrease in viability between HO12 and HO98; NS = Not Significant. Error bars = ± SEM**.**

Methods

Tumor specimen processing and organoid culture conditions. The fresh tumor specimens were collected and processed as described in our previous publication (1). Organoids were grown in PaTOM Organoid Culture media (serum-free) (2) which contains DMEM + Glutamax (Gibco; 10564-011), 0.1% Penicillin-Streptomycin-Amphotericin B Solution, 100 μg/mL Normocin (InvivoGen; ant-nr-05), 0.25 μg/mL hydrocortisone (Sigma; H0888), 1% B27 (Gibco; 12587-010), 50 μg/mL L-Ascorbic acid (Sigma; A92902), 20 μg/mL insulin (Sigma; I9278), 100 ng/mL FGF2 (R&D Systems; 233-FB), and 100 nM all-trans retinoic acid (Sigma; R2625). Organoid Culture media: PaTOM media, plus 10 μM Y-267632 (ROCK inhibitor) (Selleckchem; S1049), 5% Matrigel (Corning; 356255). Once the PDO was established, organoids were passaged as described (1). All CPM and CLM PDOs tested negative for mycoplasma using the Universal Mycoplasma Detection Kit (30-1012K; ATCC). RNA and DNA were collected during passaging for transcriptomic and genetic analysis.

Drug study protocol. For drug studies, cells isolated from PDOs by trypsinization were seeded at 5,000 cells per well in 96-well flat clear-bottom black plates (Corning; 3603) precoated with 10 μL of Matrigel. Before seeding, cells were filtered through a MACS® SmartStrainer (100 μm) filter (Miltenyi Biotec; 130-098-463) to remove large organoids. Plates were incubated overnight, and drug was added the next day. For doxorubicin, daunorubicin, idarubicin, MMC, and oxaliplatin: Drug and media were refreshed every 2-3 days. Plates were incubated for 5 days. For linsitinib and antibody combination studies: cells were treated for 24h with linsitinib (10, 50, and 100 nM), anti-IGF2 (1μg/mL or 3 μg/mL), or ganitumab (5 μg/mL or 10 μg/mL) before adding chemotherapy on day 2. Concentrations for doxorubicin (50 nM), daunorubicin (25 nM), idarubicin (6 nM), MMC (10 nM), and oxaliplatin (250 nM) were chosen since these concentrations had a small but measurable effect on viability in HO199 (10-20% reduction) as shown in Supplemental Figures 2 and 3. Antibody concentrations were chosen based on the

average IGF2 protein expression in Supplemental Figure 2J and manufacturer recommendations. Drug and media were refreshed on day 5. Plates were incubated for 6 days. For all drug studies: Each well contained an equal molar concentration of diluent (3-5 wells per condition). Plates were imaged using the Celigo Image Cytometer (Nexcelom, Lawrence, MA) on Day 0 and Day 5/6. Cell viability was measured using the CellTiter-Glo® Luminescent Cell Viability Assay (Promega; G7572). Luminescence was measured using a spectrophotometer (Molecular Devices SpectraMax® M3) and normalized relative to the control wells. The means of a minimum of 3 biological replicates were expressed relative to the control as percent cell viability. Area under the curve (AUC) was calculated based on relative cell viability using the trapezoid rule and was normalized to the maximum AUC value in Excel. Drugs used for this study included: oxaliplatin (Selleckchem; S1224), doxorubicin HCl (adriamycin) (Selleckchem; S1208), daunorubicin HCl (Selleckchem; S3035), idarubicin HCl (Selleckchem; S1228), mitomycin C (MMC) (Selleckchem; S8146), and linsitinib (OSI-906) (Selleckchem S1091). Antibodies included anti-IGF2 (R&D Systems; AF-292) and ganitumab (Selleckchem; A2456). Drugs and antibodies were prepared according to manufacturer instructions. Organoids that grew for 5 or more passages were used for drug studies.

Samples used for Drug studies, DNA-, ATAC-, RNA-Seq listed below.

Patient ID	1A	1C	1D	3A	4A	5A	6A	6B	6C	7B	10A	10C	11A	11B
Organoid ID	HO176	HO178	HO179	HO194	HO195	HO196	HO197	HO198	HO199	HO201	HO209	HO211	HO217	HO218
DNA Seq	X	X	X	X	X	X	X		X	X	X	X	X	X
ATAC Seq	X	X	X		X	X	X	X	X			X		
RNA Seq	X	X	X		X	X	X	X	X			X		
Drug Studies	X	X	X	X	X	X	X	X	X			X	X	

DNA-Seq. DNA was extracted using the Qiagen DNeasy® Blood and Tissue Kit (Qiagen; 69504). At the Mayo Clinic Genome Analysis Core, DNA concentration and quality were

determined using Qubit fluorometry (Invitrogen, Carlsbad, CA) and the Agilent Bioanalyzer (Santa Clara, CA). Libraries were prepared following the manufacturer's protocol (QIAGEN, Germantown, MD) for the QIAseq™ Targeted DNA Human Comprehensive Cancer Panel. Sixty nanograms of genomic DNA was enzymatically fragmented, end repaired, and A-tailed in a single, multi-reaction step. Enriched libraries were purified using QIAseq beads and further amplified through a universal PCR reaction in which Illumina specific adapters were added. The universal amplification reaction was purified and the final library quantified using Qubit (ThermoFisher Scientific, Waltham, MA), the Fragment Analyzer Standard Sensitivity NGS Fragment Analysis kit (Agilent, Santa Clara, CA) and the qPCR-KAPA Library Quantification Kit (Roche, Indianapolis, IN). The targeted DNA libraries were pooled and sequenced following Illumina's standard protocol using the Illumina cBot and HiSeq 3000/4000 PE Cluster Kit. The Illumina Read 1 primer was replaced with the diluted QIAseq A Read 1 Primer custom sequencing primer (original concentration at 100μM) and the flow cell was sequenced as 150 X 2 paired end reads on an Illumina HiSeq 4000 using HiSeq 3000/4000 sequencing kit and HD 3.4.0.38 collection software. Base-calling was performed using Illumina's RTA version 2.7.7

DNA-Seq Analysis. Reads from sequenced data were aligned against the Hg38 reference using bwa-mem. Variants were called using Mutect2 software. Variants were filtered and annotated using Ingenuity Variant analysis software (Qiagen) with default filters. Pathogenic and likely pathogenic variants detected with a variant allele frequency of at least 10% were considered for further analysis. PDO variants were compared to variants found in the donor tumor sequence by the Tempus Targeted Panel when available.

ATAC-seq. 100,000 cells were collected in a 1.5 mL low-binding microcentrifuge tube and brought to a total volume of 1 mL in ice-cold PBS and mixed gently. Cells were pelleted and resuspended in 100 μL of ice-cold anti-freezing buffer (10% DMSO, 10% FBS in DMEM).

Approximately 50 – 100K cells were subjected to ATAC following the Omni-ATAC protocol (3). Libraries were purified by AMPure XP magnetic beads (Beckman Coulter, A63881) and the size distribution of library DNA was determined by Fragment Analyzer (Advanced Analytical Technologies) using a High Sensitivity NGS Fragment Analysis Kit (Advanced Analytical Technologies, DNF-486). Real-time PCR analysis was performed using SYBR Green universal PCR mixes (Bio-Rad) to measure enrichment of accessible regions in the library DNAs. The following primers were used: for accessible regions, Fam86fp-F: 5'-GGCATTAGGGCGGGGATAAA-3', Fam86fp-R: 5'-TAAGACCTGTCTCCTGGGCT-3'; Ccdc146-F: 5'-GCTCCGCCTCTTAGAAACCA-3', Ccdc146-R: 5'-GCGATTCTAACACCTGCCCT-3'; for inaccessible region, hATAC-C19 inter-F: 5'-GCTTGTCTTTCCCAAGTTTACTC-3', hATAC-C19 inter-R: 5'-TAGCTGTCGCACTTCAGAGGA-3'. The libraries were sequenced to 51 base pairs from both ends on an Illumina NovaSeq 6000 SP platform in the Mayo Clinic Genome Analysis Core (3). The concentration and fragmentation profile of submitted ATAC libraries were determined using Qubit fluorometry (Invitrogen) and the Agilent Bioanalyzer. The libraries were sequenced using Illumina's standard protocol for the NovaSeq 6000. The flow cells were sequenced as 50 X 2 paired end reads using the NovaSeq SP sequencing kit and NovaSeq Control Software v1.8.0. Base-calling was performed using Illumina's RTA version 3.4.4. Libraries were combined across flow cells and trimmed using Cutadapt 1.16.5 to remove the Nextera adapters. Trimmed reads were aligned to Hg38 using Bowtie2 2.5.0 with the preset --very sensitive end-to-end. Reads were filtered for mapQuality (phred scale) ≥ 30, properly paired reads, and removal of mitochondrial reads. Duplicates were removed using Picard 2.26.10 MarkDuplicates. BAM files were converted to bigWig using Deeptools 3.3.2 bamCoverage with a bin size of 50 bases and RPKM normalization. Narrow Peaks were called using MACS2 2.2.9 Callpeak with a set extension size of 200 and a set shift size of -100. Peak detection was based on a minimum FDR cutoff of ≤0.01. Heatmaps were generated using plotHeatmap 1.012. Differential accessibility was determined with DiffBind 3.8.4 using RLE (RiP)

normalization and an FDR cutoff of ≤ 0.05. Regions enriched in ATAC-seq were annotated with GREAT version 4.0.4 using basal plus extension settings (4)(5).

RNA-seq. RNA was extracted using the Qiagen RNeasy® Micro Kit (Qiagen; 74004). At the Robarts sequencing facility, the quality of RNA was assessed using the Agilent 2100 Bioanalyzer, followed by RNA reduction, creation of indexed libraries, and sequenced on Illumina NextSeq. Libraries were sequenced using Illumina NextSeq High Output 75 cycle sequencing kits for paired-end sequencing at an average of 50M read pairs per sample using Illumina's standard protocol for the NextSeq 2000 Control Software Suite v1.4.1 and the applicable base-calling software. RNA-seq FASTQ files were aligned using STAR version 2.7.8a to the human reference genome Hg38. Gene level counts were obtained using HTseq-count v.0.9.1. Differential gene expression analysis was performed with R package DESeq2 Version 2.11.40.7. Genes with a base mean ≥1, log2 fold change >1 or <-1, and an FDR ≤ 0.05 were considered significantly differentially expressed. Heatmaps were created using the R package ggplot2 version 3.3.3.

BETA Analysis. Binding and Expression Target Analysis (BETA) analysis was done using BETA basic with Hg38 as the reference genome (window 250 kb; summit ±200 bp, blacklist filtered) (6). ATAC-seq differentially accessible regions (DARs) were identified with DiffBind using RLE (RiP) normalization and an FDR cutoff of ≤ 0.05. RNA-seq differentially expressed genes were identified using DESeq2 with a base mean ≥1, log2 fold change >1 or <-1, and an FDR ≤ 0.05.

qRT-PCR. RNA was extracted using the Qiagen RNeasy® Micro Kit (Qiagen; 74004). 0.5 µg of total RNA was reverse-transcribed using the High-Capacity cDNA Reverse Transcription Kit (Thermo Fisher Scientific, 04368813). A portion of the total cDNA was amplified by real-time PCR. Samples were prepared with iTaqTM Universal SYBR Green Supermix (Bio-Rad,

1725121) and the following primers for *IGF2* (5'-3'): GACCGCGGCTTCTACTTCA (sense) and AAGAACTTGCCCACGGGGTAT (antisense), *TBP* (5'-3'): TATAATCCCAAGCGGTTTGC (sense) and CCCAACTTCTGTACAACTCTAGCA (antisense), and *GAPDH* (5'-3'): AGGTGAAGGTCGGAGTCA (sense) and GAACATGTAAACCATGTAGTTGAGG (antisense). qPCR was performed in a CFX384 Touch Real-Time PCR Detection System (Bio-Rad) using the following thermal protocol: 50°C for 2 min, then 95°C for 10 min, followed by 40 cycles of amplification (95°C for 15 s and 60°C for 60 s). Melting curve analysis was performed in the range of 65°C to 95°C, 0.5°C per 5 s increments. Sample mRNA levels were normalized to their respective *GAPDH* and *TBP* mRNA levels. Fold change in expression was calculated using the 2ΔCp method.

Enzyme-Linked Immunosorbent Assay (ELISA). PDOs were incubated in PaTOM media at 37°C for 72 h and the supernatant was collected (including a control well coated with matrigel and PaTOM). Samples were centrifuged at 4°C 1500 RPM for 5 minutes to remove particulates. Samples were prepared for ELISA following the "Cell Culture Supernates" protocol in the Quantikine ELISA Kit for IGF-II (Bio-Techne, DG200). Protein detection was performed following the kit protocol. Optical density was measured at 450 nm and subtracted from readings at 540 nm using the SpectraMax M3 Spectrophotometer (Molecular Devices). IGF2 concentration was determined relative to the standard curve. IGF2 concentrations were normalized to the sample total protein as measured using the Pierce BCA Protein Assay Kit (Thermo Fisher Scientific, 23227).

Immunofluorescence, H&E, and immunohistochemistry. PDOs were prepared for IF as previously described (1). The slides were imaged using a Zeiss 710 Confocal Microscope. Staining of tissue was performed using the following antibodies and dilutions: anti-CDX2 (Cell

Signaling; 12306S; 1:500) and anti-CK20 (Abcam; ab76126, 1:100). Secondary Antibody was Goat anti-Rabbit IgG (H+L), Alexa Fluor 594 (Thermo Fisher Scientific; A-11037; 1:500). For the H&E stain of PDOs, slides were deparaffinized and immersed in Gill's no.1 Hematoxylin Stain 3 times, rinsed with deionized water, dipped in 0.3% acid alcohol twice, rinsed with deionized water, dipped in aqueous ammonia, washed in tap water, dipped in 80% alcohol, and then counterstained with Eosin-Y with Phloxine. The slides were dehydrated in 95% alcohol twice, 100% alcohol twice, and then xylene twice and a coverslip was applied. Slides of primary tumor were prepared for H&E staining and IHC of selected surface proteins using standard clinical procedures through the Pathology Research Core in the Department of Pathology and Laboratory Medicine (Mayo Clinic, Rochester, MN).

Highest Single Agent (HSA) analysis. For each organoid and each drug combination, we determined the mean percent viability for drug A alone, the mean percent viability for drug B alone, and the mean percent viability for the combination. The HSA difference score was defined for each biological replicate as the mean viability of the most effective single agent (i.e., the agent with the lowest viability) minus the mean viability of the combination (7). Positive HSA difference values indicate the combination reduces viability more than the HSA, while negative HSA differences indicate the combination is less effective than the HSA. HSA differences were averaged across replicates for each organoid to obtain the mean HSA score. Statistical significance was assessed by comparing the distribution of replicate level HSA difference scores for each PDO to the negative control PDO using a Wilcoxon rank sum test. A *P* value of ≤ 0.05 was considered statistically significant.

Western Blot. PDOs were seeded at 5×10^5 cells per well in a 12-well Tissue Culture plate precoated with Matrigel. After 3 days, PDOs were washed with PBS and treated with either vehicle (control), linsitinib (50 nM), or ganitumab (10 μg/μL) for 6 hours. Following treatment,

cells were washed with ice-cold PBS and Matrigel was degraded using 0.5 mL of Digestion Media (DM) for 1 hour at 37°C as described in our previous publication (1). DM was neutralized in 0.5 mL of Resuspension Media (RM) and PDOs were collected in a 1.7 mL tube. PDO suspension was pelleted and washed once in ice-cold PBS. PDOs were lysed in 100 µL of RIPA buffer (50 mM Tris-HCL [pH 7.5], 1.0% NP-40, 150 mM NaCl, 5 mM EDTA, 0.25% sodium deoxycholate detergent, and 1.0% SDS) supplemented with phosphatase inhibitors (Thermo Fisher Scientific, P178420) and cOmplete protease inhibitor cocktail (Roche, 11836145001). Cell lysates were dissociated using a 1 mL syringe and proteins concentrations were determined using the BCA protein reagent (Thermo Fisher Scientific, 23227) with a BSA standard curve. Equal amounts of protein were diluted in Laemmli buffer with 20 mg/mL DTT and ran on 7.5% precast gel (Bio-Rad, 4561024). Gels were transferred to PVDF membrane using Towbin transfer buffer. The transferred membranes were blocked in 3% BSA in TBST for 1 hour at room temperature. Membranes were incubated overnight at 4°C with primary antibody in 3% BSA. The following primary antibodies were used: phospho-S473-AKT (Cell Signaling, 4060S) and AKT (Cell Signaling, 9272). After washing in TBST, blots were incubated for 1 hour with goat anti-rabbit IgG antibody (AP132, Millipore Sigma) and washed. Immunoreactive signals on blots were detected with SuperSignal West Pico Chemiluminescent Substrate (Thermo Fisher Scientific, 34080, Thermo) and imaged using a Bio-Rad ChemiDoc MP Imaging System. For quantitative analysis, digital images were quantified using Image J gel analysis tools (v.1.49). The intensity of p-AKT signal was normalized to Total AKT and vehicle control was set to 1 for each condition.

Data Availability

Requests for resources and reagents included in this manuscript should be directed to and will be fulfilled by the corresponding author, MEFZ (fernandezzapico.martin@mayo.edu). The organoid RNA and ATAC sequencing data have been deposited at the Gene Expression

Omnibus archive (RNA = GSE294934 and ATAC = GSE295009). Values for all data points in graphs are reported in the Supporting Data Values file.

Statistics

All drug response assays performed in 3 or more biological replicates. *P* values indicate level of significance using the Wilcoxon Rank Sum test between groups. A *P* value ≤0.05 was considered significant. For all line graphs, the values reported equal the means of a minimum of 3 biological replicates expressed relative to the control as percent cell viability. Error bars in line graphs = ± SEM between biological replicates. For AUC, values derived from associated line graphs. Each dot equals one biological replicate unless otherwise noted. The error bars = ± SEM between the average AUCs for the biological replicates. The black dot within each error bar = sample mean AUC. The dashed line = cohort mean AUC.

Study Approval

Tissue samples for PDOs from cytoreductive surgery from eleven patients were collected under Mayo Clinic's Institutional Review Board (IRB) protocol 19-004196. US-biopsy of liver metastases for CLMs were collected under a separate IRB Mayo Clinic 17-003174 for the preparation of PDOs. Research biopsies were obtained after the malignant lesion(s) were located and biopsied as part of routine clinical care following receipt of written informed consent from participants. Tumor samples were sent for Tempus Targeted Sequencing (Tempus, Chicago, IL) as part of routine clinical care when possible. Following specimen collection, the laboratory assigned a lab number to the specimen and the following information was collected: patient name, clinic number, IRB number, diagnosis, tumor sample site, type of sample, and organoid initiation date. Additional information regarding the primary tumor for patients in the study is listed below.

Patient ID	1	2	3	4	5	6	7	8	9	10	11
Tumor Sidedness	Right	Left	Left	Left	Left	Left	Right	Right	Right	Left	Right
Tumor Features	Mucin	Adeno	Adeno	Adeno	Adeno	Adeno	Adeno	Mucin	Adeno	Adeno	Adeno
Tumor DNA Sequenced	X			X	X	X	X			X	X

Adeno = Adenocarcinoma
Mucin = Mucinous

Sex as a Biological Variable

This study included samples derived from both male and female patients. The study was not powered to assess sex-specific effects; therefore, sex was not analyzed as a biological variable.

Author Contributions

TLH conducted experiments, performed overall experimental design, optimized relevant protocols, bioinformatics analysis, and wrote the manuscript with MDT and MFZ. WJP assisted with experiment design, protocol optimization, and organoid proliferation. ZSP and LLA optimized organoid proliferation protocols and assisted with drug studies in the organoids. JMH and TEG prepared clinical protocols and coordinated obtaining patient samples. HX, JJL, and RMC extracted and reviewed patient clinical information. MTB and EJ assisted in the bioinformatics analysis of the organoids. REV and DLM critically read the manuscript and reviewed all statistical analyses.

References

1. Hogenson TL, Xie H, Phillips WJ, Toruner MD, Li JJ, Horn IP, et al. Culture media composition influences patient-derived organoid ability to predict therapeutic responses in gastrointestinal cancers. *JCI Insight.* 2022;7(22).
2. Huang L, Holtzinger A, Jagan I, BeGora M, Lohse I, Ngai N, et al. Ductal pancreatic cancer modeling and drug screening using human pluripotent stem cell- and patient-derived tumor organoids. *Nat Med.* 2015;21(11):1364–71.
3. Corces MR, Trevino AE, Hamilton EG, Greenside PG, Sinnott-Armstrong NA, Vesuna S, et al. An improved ATAC-seq protocol reduces background and enables interrogation of frozen tissues. *Nat Methods.* 2017;14(10):959–62.
4. McLean CY, Bristor D, Hiller M, Clarke SL, Schaar BT, Lowe CB, et al. GREAT improves functional interpretation of cis-regulatory regions. *Nat Biotechnol.* 2010;28(5):495–501.
5. Tanigawa Y, Dyer ES, and Bejerano G. WhichTF is functionally important in your open chromatin data? *PLoS Comput Biol.* 2022;18(8):e1010378.
6. Wang S, Sun H, Ma J, Zang C, Wang C, Wang J, et al. Target analysis by integration of transcriptome and ChIP-seq data with BETA. *Nat Protoc.* 2013;8(12):2502–15.
7. Berenbaum MC. What is synergy? *Pharmacol Rev.* 1989;41(2):93–141.
