# Supplementary material for: Epigenomic profiling links IGF2 overexpression to anthracycline resistance in colorectal cancer organoids
Source: JCI Insight. 2026 Jul 16;11(16):e195194. doi: 10.1172/jci.insight.195194 (PMC13505381; doi:10.1172/jci.insight.195194)
Supplement: Supplementary file 2 [file jciinsight-11-195194-s003.pdf]

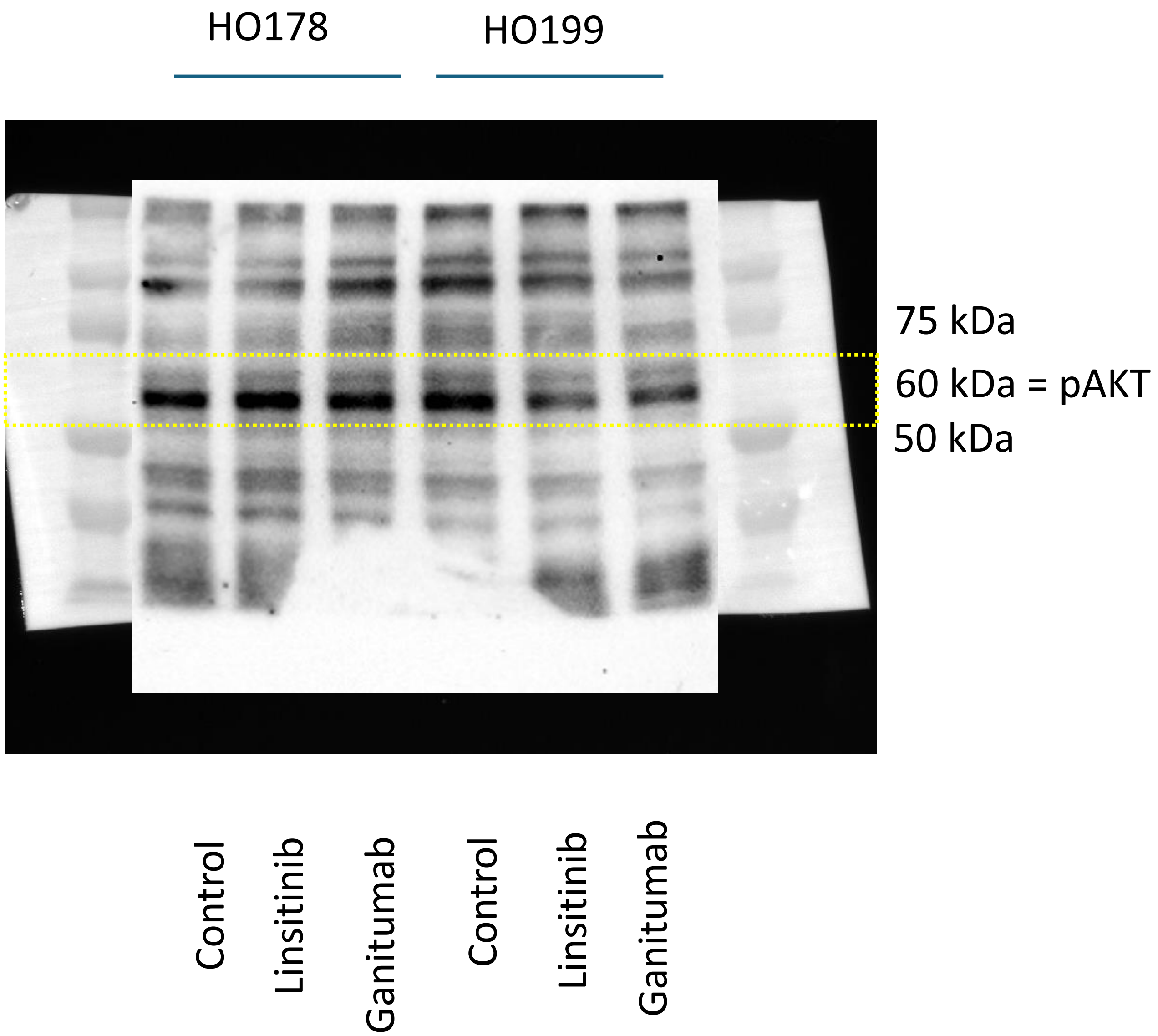
HO178
HO199
75 kDa
60 kDa = pAKT
50 kDa
Control
Linsitinib
Ganitumab
Control
Linsitinib
Ganitumab

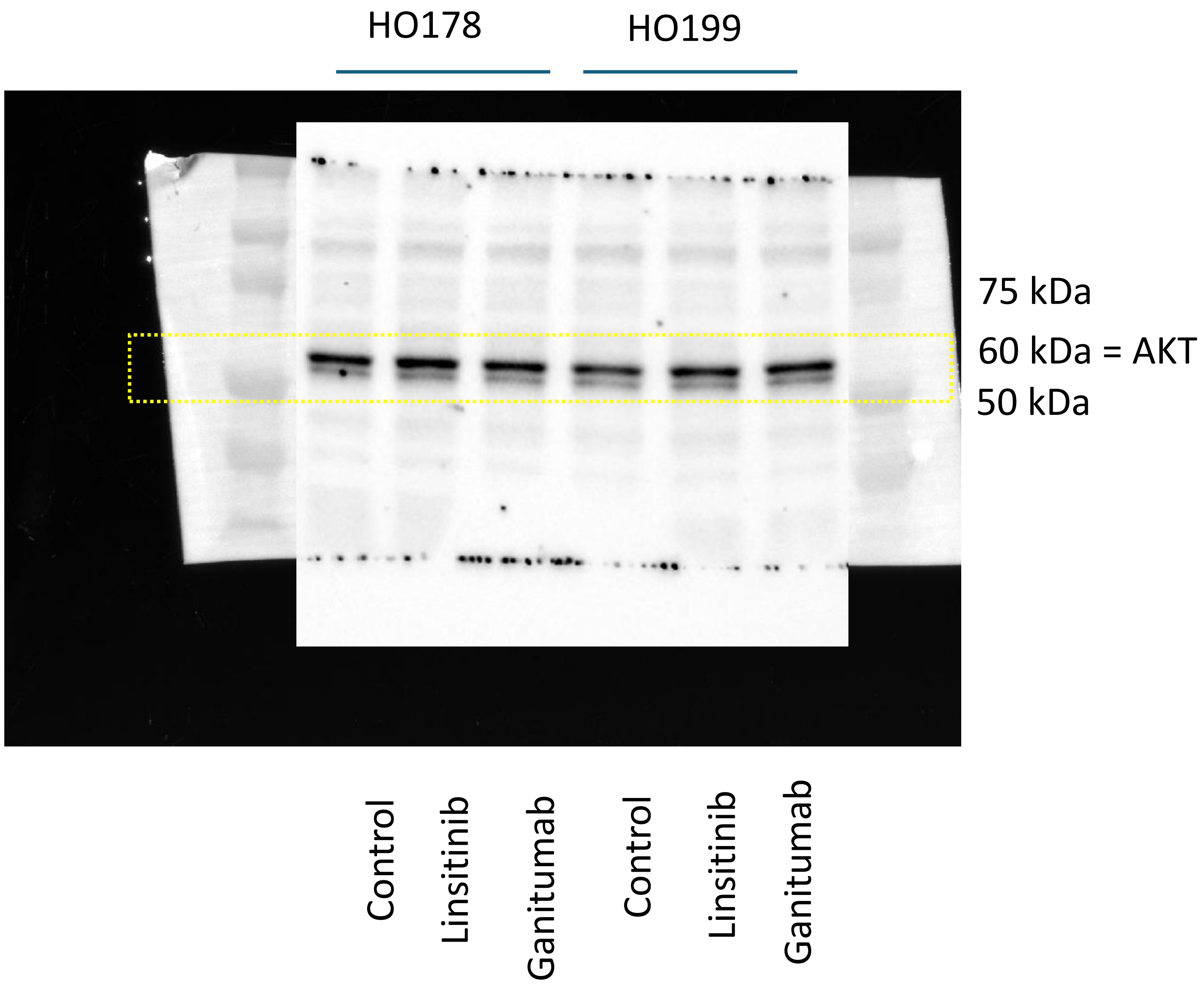
HO178
HO199
75 kDa
60 kDa = AKT
50 kDa
Control
Linsitinib
Ganitumab
Control
Linsitinib
Ganitumab
